# Progress Toward Measles Elimination — Western Pacific Region, 2013–2017

**DOI:** 10.15585/mmwr.mm6717a3

**Published:** 2018-05-04

**Authors:** José E. Hagan, Jennifer L. Kriss, Yoshihiro Takashima, Kayla Mae L. Mariano, Roberta Pastore, Varja Grabovac, Alya J. Dabbagh, James L. Goodson

**Affiliations:** ^1^Western Pacific Regional Office, Expanded Programme on Immunization, World Health Organization, Manila, Philippines; ^2^Global Immunization Division, Center for Global Health, CDC; ^3^Department of Immunization, Vaccines, and Biologicals, World Health Organization, Geneva, Switzerland.

In 2005, the Regional Committee for the World Health Organization (WHO) Western Pacific Region (WPR)* established a goal for measles elimination^†^ by 2012 (*1*). To achieve this goal, the 37 WPR countries and areas implemented the recommended strategies in the WPR Plan of Action for Measles Elimination (*2*) and the Field Guidelines for Measles Elimination (*3*). The strategies include 1) achieving and maintaining ≥95% coverage with 2 doses of measles-containing vaccine (MCV) through routine immunization services and supplementary immunization activities (SIAs), when required; 2) conducting high-quality case-based measles surveillance, including timely and accurate testing of specimens to confirm or discard suspected cases and detect measles virus for genotyping and molecular analysis; and 3) establishing and maintaining measles outbreak preparedness to ensure rapid response and appropriate case management. This report updates the previous report (*4*) and describes progress toward measles elimination in WPR during 2013–2017. During 2013–2016, estimated regional coverage with the first MCV dose (MCV1) decreased from 97% to 96%, and coverage with the routine second MCV dose (MCV2) increased from 91% to 93%. Eighteen (50%) countries achieved ≥95% MCV1 coverage in 2016. Seven (39%) of 18 nationwide SIAs during 2013–2017 reported achieving ≥95% administrative coverage. After a record low of 5.9 cases per million population in 2012, measles incidence increased during 2013–2016 to a high of 68.9 in 2014, because of outbreaks in the Philippines and Vietnam, as well as increased incidence in China, and then declined to 5.2 in 2017. To achieve measles elimination in WPR, additional measures are needed to strengthen immunization programs to achieve high population immunity, maintain high-quality surveillance for rapid case detection and confirmation, and ensure outbreak preparedness and prompt response to contain outbreaks.

## Immunization Activities

MCV1 and MCV2 coverage data are reported annually to WHO and the United Nations Children’s Fund (UNICEF) from 36 of the 37 WPR countries and areas.^§^ WHO and UNICEF estimate vaccination coverage for 27 countries/areas in the region, using annual government-reported survey and administrative data; for the remaining areas and territories, reported coverage data from immunization program monitoring are used. Regional MCV1 and MCV2 coverage rates were maintained at ≥95% and >90%, respectively, during 2013–2016 (Table 1). In 2016, 18 (50%) of 36 countries achieved ≥95% MCV1 coverage, and 11 (31%) reported ≥95% coverage with both MCV1 and MCV2. As of 2017, only two (5%) WPR countries and areas (Solomon Islands and Vanuatu) had not yet introduced MCV2. During 2013–2017, 18 national SIAs^¶^ were conducted (Table 2); in addition, Japan conducted annual SIAs targeting schoolchildren aged 13 years and 17 years. Reported vaccination coverage was ≥95% in seven (39%) of the nationwide SIAs.

**TABLE 1:** Measles-containing vaccine (MCV) schedule, estimated coverage with the first and second dose of MCV,* number of confirmed measles cases,^†^ and confirmed measles incidence, by country/area — World Health Organization Western Pacific Region, 2013, 2016, and 2017

Country/Area	MCV schedule^§^		2013				2016				2017^¶^	
			Coverage (%)		No. of measles cases	Incidence per million population	Coverage (%)		No. of measles cases	Incidence per million population	No. of measles cases	Incidence per million population
	Age when 1st dose given	Age when 2nd dose given	MCV1	MCV2			MCV1	MCV2				
American Samoa**	12 mos	4 yrs	NR^††^	NR^††^	0	0	NR^††^	NR^††^	0	0.0	0	0.0
Australia	12 mos	18 mos	94	92	154	6.7	95	94	99	4.1	81	3.3
Brunei	12 mos	18 mos	96	92	0	0.0	98	97	1	2.4	0	0.0
Cambodia	9 mos	18 mos	76	49	0	0.0	81	58	56	3.6	10	0.6
China	8 mos	18 mos–24 mos	99	99	27,825	20.1	99	99	24,839	17.7	5,993	4.3
CNMI**	12 mos	4 yrs	68^§§^	65^§§^	0	0.0	62	72	0	0.0	0	0.0
Cook Islands	15 mos	4 yrs	97	95	0	0.0	90	90	0	0.0	0	0.0
Fiji	12 mos	6 yrs	94	94	0	0.0	94	94	5	5.6	1	1.1
French Polynesia**	12 mos	18 mos	99^§§^	98^§§^	0	0.0	99^§§^	98^§§^	0	0.0	0	0.0
Guam**	12 mos	4 yrs–6 yrs	51^§§^	44^§§^	0	0.0	92	NR^††^	0	0.0	0	0.0
Hong Kong (China)**	12 mos	6 yrs	95	95	38	5.3	95	95	9	1.2	4	0.5
Japan	12 mos	5 yrs	95	93	207	1.6	96	93	157	1.2	187	1.5
Kiribati	12 mos	6 yrs	91	84	0	0.0	80	79	0	0.0	0	0.0
Laos^¶¶^	9 mos	12 mos	82	NA***	68	10.5	76	NA***	8	1.2	3	0.4
Macao (China)**	12 mos	18 mos	99	96	3	5.2	94	92	0	0.0	2	3.2
Malaysia	12 mos	7 yrs	95	99	182	6.1	96	99	1,587	50.9	1,648	52.1
Marshall Islands	12 mos	13 mos	79	56	0	0.0	75	49	0	0.0	0	0.0
Micronesia	12 mos	13 mos	91	75	0	0.0	70	74	0	0.0	0	0.0
Mongolia	9 mos	2 yrs	97	97	0	0.0	98	90	28,813	9,517.4	9	2.9
Nauru	12 mos	15 mos	97	88	0	0.0	98	96	0	0.0	0	0.0
New Caledonia**	12 mos	16 mos	96	86	0	0.0	96	86	0	0.0	0	0.0
New Zealand	15 mos	4 yrs	92	86	25	5.5	92	89	104	22.3	15	3.2
Niue	15 mos	4 yrs	99	99	0	0.0	99	99	0	0.0	0	0.0
Palau	12 mos	15 mos	99	98	0	0.0	96	95	0	0.0	0	0.0
Papua New Guinea	9 mos^†††^	18 mos	89	NR^††^	9	1.2	70	NR^††^	0	0.0	7	0.8
Philippines	9 mos	12 mos–15 mos	87	54	5,798	58.9	80	66	641	6.2	1,224	11.7
Samoa	12 mos	15 mos	90	72	0	0.0	68	44	0	0.0	0	0.0
Singapore	12 mos	15 mos–18 mos	95	90	66	12.3	95	88	157	27.9	80	14.0
Solomon Islands	12 mos	NA***	93	NA***	0	0.0	99	NA***	1	1.7	0	0.0
South Korea	12 mos–15 mos	4 yrs–6 yrs	99	95	107	2.1	98	97	18	0.4	7	0.1
Tokelau**	12 mos	15 mos	100	100	0	0.0	100^§§^	100^§§^	0	0.0	0	0.0
Tonga	12 mos	18 mos	86	86	0	0.0	84	85	0	0.0	0	0.0
Tuvalu	12 mos	18 mos	96	84	0	0.0	96	92	0	0.0	0	0.0
Vanuatu	12 mos	NA***	53	NA***	0	0.0	53	NA***	0	0.0	0	0.0
Vietnam	9 mos	18 mos	98	86	1,232	13.5	99	95	368	3.9	667	7.0
Wallis and Futuna**	12 mos	18 mos	>100^§§^	>100^§§^	0	0.0	79^§§^	80^§§^	0	0.0	0	0.0
**Western Pacific Region**	**—**	**—**	**97**	**91**	**35,700**	**19.2**	**96**	**93**	**56,836**	**30.1**	**9,938**	**5.2**

**Abbreviations:** CNMI = Commonwealth of the Northern Mariana Islands; MCV1 = first dose of MCV; MCV2 = second dose of MCV; WHO = World Health Organization.

* WHO-United Nations Children's Fund (UNICEF) estimates.

^†^ Includes confirmed cases by laboratory or epidemiologic linkage and clinically compatible cases meeting the WHO clinical case definition of measles for which no adequate specimen was collected and that cannot be epidemiologically linked to a laboratory-confirmed case of measles.

^§^ MCV schedule is the 2017 schedule.

^¶^ 2017 MCV1 and MCV2 coverage estimates not available.

** Country or area reported coverage for MCV1 and MCV2 based on administrative data.

^††^ NR = not reported (country did not report coverage in the year specified).

^§§^ No data available for assessment year; data from previous year is reported instead.

^¶¶^ Laos introduced MCV2 in 2017.

*** NA = not applicable (dose was not included in the vaccination schedule for that year).

^†††^ Additional 6-month dose provided nationally.

**TABLE 2:** Characteristics of measles supplementary immunization activities (SIAs),* by year and country/area — World Health Organization Western Pacific Region, 2013–2017

Year	Country/Area	Age group targeted	Vaccine used	Extent of SIA	No. (%) of population reached in targeted age group
2013	Cambodia	9 mos–14 yrs	MR	National	4,576,633 (>100)
	Federated States of Micronesia	12 mos–47 mos	MMR	Subnational	3,435 (95)
	Philippines	6 mos–59 mos	M	National	1,937,471 (ND)
	Singapore	6 yrs–7 yrs	MMR	National	38,436 (95)
	Vanuatu	12 mos–59 mos	MMR	National	33,604 (>100)
	Vietnam	1 yr–15 yrs	M	Subnational	163,870 (94)
2014	Federated States of Micronesia	6 mos–57 yrs^†^	MMR	National	71,388 (87)
	Laos	9 mos–9 yrs	MR	National	1,569,613 (100)
	Malaysia	6 mos–17 yrs	M	Subnational	54,656 (63)
	Philippines	9 mos–59 mos	MR	National	10,402,489 (91)
	Philippines	6 mos–36 mos	M	Subnational	1,695,930 (78)
	Vietnam	9 mos–24 mos	M	National	875,386 (94)
2015	Malaysia	9 mos–17 yrs	MMR	Subnational	21,518 (90)
	Mongolia	9 mos–17 yrs	M	National	347,685 (94)
	Papua New Guinea	9 mos–14 yrs	MR	National	801,436 (62)
	Vanuatu	6 mos–59 mos	M	Subnational	24,336 (98)
	Vanuatu	1 yr–15 yrs	MR	National	103,676 (>100)
	Vietnam	1 yr–14 yrs	MR	National	19,740,181 (98)
2016	Cambodia	9 mos–59 mos	MR	National	766,743 (91)
	Malaysia	1 yr–17 yrs	MR	Subnational	139,954 (85)
	Mongolia	18 yrs–30 yrs	MR	National	549,846 (88)
	Papua New Guinea	9 mos–15 yrs	MR	Subnational	436,854 (63)
	Vietnam	16 yrs–17 yrs	MR	National	1,787,588 (95)
2017	Cambodia	6 mos–59 mos	MR	National	1,452,821 (75)
	Fiji	12 mos–11yrs	MR	National	ND
	Laos	9 mos–4 yrs	MR	National	ND
	Papua New Guinea	6 mos–45 yrs	MR	Subnational	ND
**2013–2017**	**Western Pacific Region**				**47,595,549 (93^§^)**

**Abbreviations:** M = monovalent measles vaccine; MMR = measles, mumps, and rubella vaccine; MR = measles and rubella vaccine; ND = no data.

* SIAs generally are carried out using two approaches. An initial, nationwide catch-up SIA targets all children aged 9 months–14 years; it has the goal of eliminating susceptibility to measles in the general population. Periodic follow-up SIAs then target all children born since the last SIA. Follow-up SIAs generally are conducted nationwide every 2–4 years and generally target children aged 9–59 months; their goal is to eliminate any measles susceptibility that has developed in recent birth cohorts and to protect children who did not respond to the first measles vaccination. The exact age range for follow-up SIAs depends on the age-specific incidence of measles, coverage with measles-containing vaccine through routine services, and the time since the last SIA.

^†^ Targeted age groups varied by province.

^§^ Average SIA coverage, weighted by size of target population.

## Surveillance Activities

Case-based measles and rubella surveillance data are reported monthly to WHO from all WPR countries and areas; 21 countries and areas of the Pacific Islands report data as one epidemiologic block.** The WHO Global Measles and Rubella Laboratory Network supports surveillance by providing laboratory confirmation and genotyping of reported cases. Suspected measles cases are confirmed based on laboratory findings, an epidemiologic link, or clinical criteria.^††^ Key indicators of surveillance performance include 1) the number of suspected measles cases discarded as nonmeasles (target: ≥2 per 100,000 population); 2) the proportion of second-level administrative units with two or more nonmeasles discarded cases per 100,000 population (target: ≥80%); 3) the percentage of suspected measles cases with adequate investigation that includes all essential data elements^§§^ (target: ≥80%); 4) the percentage of suspected measles cases with adequate specimens collected within 28 days of rash onset (target: ≥80%, excludes epidemiologically linked cases); and 5) the percentage of specimens with laboratory results available within 7 days after receipt in the laboratory (target: ≥80%). During 2013–2017, the number of WPR countries and areas^¶¶^ that met the target for suspected cases discarded as nonmeasles per 100,000 population at the national level decreased from 11 (65%) to nine (53%), but increased from one (6%) to two (12%) at the subnational level. From 2013 to 2017, the percentage of suspected cases with adequate investigations decreased from 92% to 89%; the percentage of suspected cases with adequate specimens collected for laboratory testing decreased from 90% to 89%; and the proportion of blood specimens received by the laboratory with results available within 7 days increased from 84% to 98% (Supplementary Table, https://stacks.cdc.gov/view/cdc/53519).

## Measles Incidence and Genotypes

WPR experienced a resurgence of measles during 2013–2016 (Figure), after a record low incidence of 5.9 cases per million population in 2012. During the resurgence, incidence of seasonal endemic measles virus transmission in China increased, and large-scale nationwide outbreaks occurred in other countries with endemic measles (Malaysia and the Philippines). During 2013–2016, after importations from countries with endemic disease, measles outbreaks also occurred in countries that had been verified as having eliminated endemic measles virus transmission (Australia, Cambodia, Japan, and South Korea), including a large-scale outbreak in Mongolia. An increase in importations also led to outbreaks in several countries with endemic, low-incidence measles, including New Zealand, Papua New Guinea, Singapore, Solomon Islands, and Vietnam. Annual regional measles incidence per 1 million population increased from 19.2 in 2013 to 68.9 in 2014, and then decreased to 5.2 in 2017, a historic low (Table 1). The predominantly detected circulating measles virus genotypes were H1 in China, B3 in the Philippines, and both D8 and D9 in Malaysia and Vietnam.

**FIGURE:**
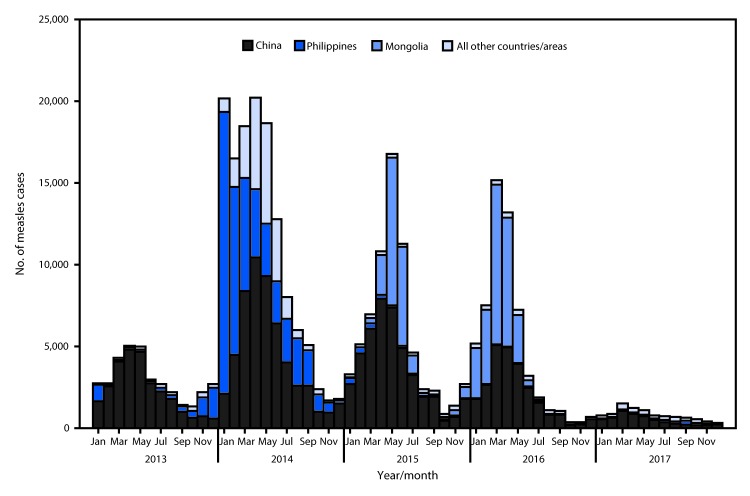
Confirmed measles cases,* by month of rash onset — World Health Organization Western Pacific Region, 2013–2017 * Confirmed and clinically compatible measles cases reported by countries and areas to the World Health Organization (WHO). A case of measles was laboratory-confirmed when measles-specific immunoglobulin M antibody was detected in serum, measles-specific RNA was detected by polymerase chain reaction, or measles virus was isolated in cell culture from a person who was not vaccinated during the 30 days before rash onset. A case of measles was confirmed by epidemiologic linkage when linked in time and place to a laboratory-confirmed measles case without serologic confirmation. During 2013–2017, a case of measles meeting the WHO clinical case definition but without a specimen collected could be reported as clinically compatible.

## Regional Verification of Measles Elimination

After the request of the Western Pacific Regional Committee (*5*) for WHO to establish a formal mechanism for verification of elimination through the Regional Verification Commission, verification guidelines were finalized in April 2013 and revised in 2016 to include verification of rubella elimination. As of the September 2017 Regional Verification Commission meeting, a total of eight (47%) WPR countries and areas (Australia, Brunei, Cambodia, Hong Kong [China], Japan, Macao [China], New Zealand, and South Korea) have been verified as having achieved elimination of measles (*6*). After a nationwide outbreak in Mongolia during 2015–2016 that lasted longer than 12 months, the Regional Verification Commission determined that endemic measles virus transmission had been reestablished in Mongolia (*7*).

## Discussion

The 2013–2016 measles resurgence in WPR was attributed to three factors. First, increased measles virus transmission occurred in countries with endemic disease (e.g., China, Malaysia, and the Philippines). Second, large-scale outbreaks occurred after importation of measles into countries with endemic, low-incidence measles. Third, multiple measles importations into countries or areas that had achieved elimination occurred, particularly in Mongolia, where a large outbreak persisted for >12 months and endemic measles virus transmission was reestablished.

Measles incidence in WPR declined to a historic low of 5.2 in 2017 because of achievement of control of the outbreaks in Vietnam (2013–2014) and Mongolia (2015–2016), burnout of the outbreak in the Philippines (2013–2014), and China’s accelerated control of measles after the 2010–2011 outbreak. However, the resurgence during 2013–2016 revealed ongoing and emerging challenges that need to be addressed. These challenges include changing measles epidemiology, with increased measles incidence occurring among adolescents, young adults, and infants too young to be vaccinated as well as heterogeneity of measles epidemiology among subnational areas and specific groups at risk within countries with large populations. In addition, the resurgence revealed systems weaknesses: immunization programs were unable to achieve and maintain high population immunity through routine immunization service delivery, and some national laboratories had insufficient capacity to conduct timely serologic testing during outbreaks. The challenges identified also included inadequately developed and implemented policies and processes for preventing measles resurgence and morbidity after virus introduction to the population, including delayed outbreak investigation, insufficient outbreak response, and nosocomial measles virus transmission. Finally, the resurgence revealed a need for greater involvement of local governments, private sectors, societies, and communities.

To address these challenges and to accelerate measles elimination efforts in WPR, the WHO Regional Office for the Western Pacific prepared a new strategy and plan of action for measles and rubella elimination in the Western Pacific that was endorsed by the 68th meeting of the WHO Regional Committee for the Western Pacific in October 2017 (*5*). The document details 31 strategies with accompanying activities in the following eight areas: 1) overall planning; 2) immunization services; 3) epidemiologic surveillance; 4) laboratory support; 5) program review and risk assessment; 6) outbreak preparedness and response; 7) partnerships, advocacy, information, education and communication, and social mobilization; and 8) progress monitoring and verification of elimination. The new regional strategy is designed to address specific challenges facing WPR countries and to serve as a resource for development of national plans of action (and subnational plans for countries with large populations), tailored to country-specific opportunities for achieving and maintaining measles elimination.

Collective efforts among WPR countries are important for achieving regional measles elimination. Working together to follow the recommended strategies and actions in the Regional Strategy and Plan of Action for Measles and Rubella Elimination could help WPR countries in their efforts to strengthen immunization programs to achieve and sustain high population immunity, maintain high-quality surveillance for rapid case detection and confirmation, and ensure outbreak preparedness and prompt response to contain outbreaks.

**Summary**

**What is already known about this topic?**

Most countries in the World Health Organization Western Pacific Region (WPR) have made substantial progress toward measles elimination.

**What is added by this report?**

During 2013–2016, a resurgence of measles occurred in WPR, with large-scale outbreaks in Mongolia, the Philippines, and Vietnam, and increased endemic transmission in China; in 2014, annual incidence increased to 68.9 cases per million. However, with control of the outbreaks, in 2017, incidence decreased to a new historic low (5.2 per million).

**What are the implications for public health practice?**

Achieving high reported vaccination coverage is not sufficient for achieving regional measles elimination. Efforts by WPR countries are needed to establish high population immunity, build strong immunization systems, maintain high-quality surveillance, and improve outbreak preparedness and response.

## Footnotes

* The Western Pacific Region, one of the six regions of the World Health Organization, consists of 37 countries and areas with a population of approximately 1.8 billion, including American Samoa (USA), Australia, Brunei, Cambodia, China, Commonwealth of the Northern Mariana Islands, Cook Islands, Federated States of Micronesia, Fiji, French Polynesia (France), Guam (USA), Hong Kong (China), Japan, Kiribati, Laos, Macao (China), Malaysia, Marshall Islands, Mongolia, Nauru, New Caledonia (France), New Zealand, Niue, Palau, Papua New Guinea, Philippines, Pitcairn Islands (UK), Samoa, Singapore, Solomon Islands, South Korea, Tokelau (New Zealand), Tonga, Tuvalu, Vanuatu, Vietnam, and Wallis and Futuna (France).

† Measles elimination is defined as the absence of endemic measles virus transmission in a defined geographical area (e.g., region or country) for ≥12 months in the presence of a well-performing surveillance system.

§ The Pitcairn Islands, with a population of approximately 50 persons, does not report immunization coverage data to WHO/UNICEF.

¶ SIAs are generally carried out using two target age ranges. An initial, nationwide catch-up SIA targets children aged 9 months–14 years, with the goal of eliminating susceptibility to measles in the general population. Periodic follow-up SIAs then target children born since the last SIA. Follow-up SIAs are generally conducted nationwide every 2–4 years and generally target children aged 9–59 months; their goal is to eliminate any measles susceptibility that has developed in recent birth cohorts and to protect children who did not respond to the first measles vaccination.

** The epidemiologic block of countries and areas of the Pacific Islands includes American Samoa, Commonwealth of the Northern Mariana Islands, Cook Islands, Federated States of Micronesia, Fiji, French Polynesia, Guam, Kiribati, the Marshall Islands, Nauru, New Caledonia, Niue, Palau, Pitcairn Islands, Samoa, Solomon Islands, Tokelau, Tonga, Tuvalu, Vanuatu, and Wallis and Futuna.

†† Cases that meet the WHO clinical case definition for measles for which no adequate specimen was collected and cannot be epidemiologically linked to a laboratory-confirmed case of measles. Before 2013, in WPR these cases were classified as "clinically confirmed." Beginning in 2013, this classification was renamed "clinically compatible" and cases were only classified "confirmed" if they were laboratory-confirmed or epidemiologically linked. The change in terms reflects the recognition in WPR that clinically compatible cases represent a weakness in surveillance.

§§ Essential data elements include name or identifier, date of birth or age, sex, place of residence, vaccination status or date of last vaccination, date of rash onset, date of notification, date of investigation, date of specimen collection, and place of infection or travel history.

¶¶ Percentages were calculated using a denominator of 17 (16 countries or areas, plus the epidemiologic block of the Pacific Islands countries and areas).
